# Supplementary material for: Non-spherical particles in optical tweezers: A numerical solution
Source: PLoS One. 2019 Dec 5;14(12):e0225773. doi: 10.1371/journal.pone.0225773 (PMC6894773; doi:10.1371/journal.pone.0225773)
Supplement: Attachment — Submitted filename: myReview.pdf [file pone.0225773.s003.pdf]

Manuscript Comments

Grammar and Spelling

The points below are indicative and non-exhaustive.

- 7: "are considered, such as in [3]" -> "are considered [3]"
- 7: "due to that dynamical modeling of the" -> "due to the fact that the dynamical modeling of"
- 8: "tweezers requires" -> "tweezers require"
- 10: "for considering" -> "to solve for"
- 11: "Frameworks" -> "Existing frameworks deal with"
- 15: "has fittingly" -> "has previously been"
- 16: "about simulating" -> "related to the simulation of"
- 20: "methodology" -> "methodology presented"
- 21: "exact volume integral equation solution" -> "exact solution of the volumen integral equation"
- 22: "is indifferent" -> "is insensitive"
- 27: "on a category" -> "on the category"
- 29-30: "we introduce a numerical.." -> "we introduce a model for numerical studies of optical tweezers for arbitarily shaped rigid particles. These have been shown experimentally"
- 47: "as a rigid tetrahedral mesh" -> "as rigid tetrahedral meshes"
- 51: "nor geometric" -> "nor the assumptions of geometric"
- 53: "deformed ellipsoidal" -> "deformed ellipsoidal particles"
- 58: "is in" -> "for"
- 69: "indeed require careful adoption" -> "need to be adapted carefully"
- 98: "In optical tweezers," -> "When considering classical optical tweezer theory,"
- 130: "forcing on" -> "forces acting on"
- 130: "tweezers is" -> "tweezers are"
- 137: "forcing" -> "forces"
- 138: "the exact" -> "this exact"
- 139: "the Brownian force is the" -> "the Brownian force is accounted for by the"
- 142: "force direction" -> "force vector"

Clarifications

- 8: "repeated solution" is unclear. Do the authors mean a recursive solution is required?
- 91: Citation required
- 103: Citation required
- 106: The mechanical interactions are unclear.
- 103-104: The authors have not given enough background on the background of the analytic spherical particle results used, and the rational for accounting for the macroscopic flow properties in terms of their order-of-magnitude estimates should be described further.
- 126-131: Citations required

- 140-141: The reference, [22] mentions "Given that We will consider a silica microparticle in water with radius R = 1 μm, mass m = 11 pg, viscosity η=0.001 Ns/m2, γ=6πηR, temperature T = 300 K, and τ=0.6 μs. We remark that τ is orders of magnitude smaller than the time scales of typical experiments" The authors should comment on how the differences in the systems considered are accounted for.
- 150-154: It is unclear how the Brownian forces are taken into account and the discussion should be left to section 3.3.
- The trapping detail does not cover if situations where multiple particles enter the trap, and the orientation and scattering should be tested against experimental studies [Mondal, Dipankar, Anushka Jha, Yogesh M. Joshi, and Debabrata Goswami. "Microrheology Study of Aqueous Suspensions of Laponite using Femtosecond Optical Tweezers." In Optical Trapping Applications, pp. OtW2E-1. Optical Society of America, 2017.]
- 318-331: Implementation details of the Brownian motion should be added, along with where it ties into the data made available. References are required to show significant Brownian motion in air, being as it is primarily a liquid-state effect.
- 381-387: Given that the VSWF expansion has not been shown mathematically for different beam types this section should be reworked to describe that the results are heuristically determined from the numerical simulations.
- Section 4 needs references from the existing literature.

General Comments

- The work presented is coherent and adds to the existing literature on te numerical simulation of particles in optical tweezers, while also providing physical insights into the tweezing process itself. The authors have considered the full scattering solution of Laguerre-Gaussian beams with the vector spherical wave function expansion.
- As PLOS ONE has no copyediting services, at this stage the manuscript should be revised for clarity and language (as per the points above). Furthermore, the points below should be addressed.

1. The authors should mention that the optical tweezers considered are for continuous wave (CW) lasers and perhaps add to how they expect pulsed laser systems to behave. The work of [De, Arijit Kumar, Debjit Roy, Aveek Dutta, and Debabrata Goswami. "Stable optical trapping of latex nanoparticles with ultrashort pulsed illumination." Applied Optics 48, no. 31 (2009): G33-G37] and [Deng, Jian-liao, Qing Wei, Yu-zhu Wang, and Yong-qing Li. "Numerical modeling of optical levitation and trapping of the “stuck” particles with a pulsed optical tweezers." Optics express 13, no. 10 (2005): 3673-3680] should be mentioned in terms of pulsed lasers.
2. The data availability is slightly unclear. The software itself is located at the Github repository and with the appropriate license, however the data used for this study is not evidently a part of this manuscript or the supporting materials. Please clarify this.
